# Phosphorescence of thermally altered human bone

**DOI:** 10.1007/s00414-020-02455-1

**Published:** 2020-11-19

**Authors:** Tristan Krap, Loes Busscher, Roelof-Jan Oostra, Maurice C. G. Aalders, Wilma Duijst

**Affiliations:** 1 Maastricht University, Maastricht, The Netherlands; 2 Department of Medical Biology, Section Anatomy, Amsterdam University Medical Centre, Location Academic Medical Centre, Meibergdreef 15, 1105 AZ Amsterdam, The Netherlands; 3 Ars Cognoscendi Foundation for Legal and Forensic Medicine, Wezep, The Netherlands; 4 Department of Life Sciences and Technology–Biotechnology–Forensic Science, Van Hall Larenstein, University of Applied Sciences, Leeuwarden, The Netherlands; 5 Department of Biomedical Engineering and Physics, Amsterdam University Medical Centre, Location Academic Medical Centre, Amsterdam, The Netherlands; 6 Co van Ledden Hulsebosch Center, Amsterdam, The Netherlands

**Keywords:** Phosphorescence, Photoluminescence, Bone, Heat, Cremation, Forensic anthropology

## Abstract

Bone has photoluminescent characteristics that can aid the analysis of thermally altered human skeletal remains as part of the forensic anthropological investigation. Photoluminescence stands collectively for fluorescence and phosphorescence. Because the difference in lifetime between fluorescence and phosphorescence is usually in the range of nano- to microseconds, it is only possible to visually determine whether bone phosphoresces when the lifetime is long enough to be observed. For this study, a distinction was made between long-decay and short-decay phosphorescence. So far, it was unknown whether (thermally altered) human bone emits long-decay phosphorescence after being illuminated and, thus, whether phosphorescence contributes to the observed photoluminescence. If so, whether the observable phosphorescence is dependent on temperature, exposure duration, surrounding medium, bone type, skeletal element, and excitation light and could aid the temperature estimation of heated bone fragments. In this study, bone samples were subjected to heat in the range of from room temperature to 900 °C for various durations in either air or adipose as surrounding medium. In addition, different skeletal elements of a human cadaver were recollected after cremation in a crematorium. Both sample collections were illuminated with light of different bandwidths and visually inspected for phosphorescence and photoluminescence. The samples were scored by means of a scoring index for the intensity of long-decay phosphorescence and photographically documented. The results show that thermally altered human bone fragments do phosphoresce. The observed phosphorescence is more dependent on temperature than on exposure duration, surrounding medium or skeletal element. Of the used wavelength bands, ultraviolet light provided the most temperature-related information, showing changes in both phosphorescence intensity and emission spectrum. Long-decay phosphorescence and fluorescence with short-decay phosphorescence coincide; however, there are also temperature-dependent differences. It is therefore concluded that phosphorescence contributes to the observable photoluminescence and that the visibly observable phosphorescent characteristics can aid the temperature estimation of cremated human skeletal fragments.

**Supplementary Information:**

The online version contains supplementary material available at 10.1007/s00414-020-02455-1.

## Introduction

Thermally altered human bone has photoluminescent characteristics [1–5]. These characteristics can aid the search for human skeletal remains in difficult contexts. The contrast created between the debris and the osseous material by illumination makes quick differentiation possible. Next to the retrieval of human skeletal remains, the photoluminescent characteristics can also be helpful when interpreting the degree of thermal destruction and thereby indicating the temperature that bone has been subjected to [1, 3]. The estimated temperature that bone reached during a fire can be used to reconstruct the peri- and postmortem events and to substantiate the decision on whether samples are still eligible for molecular analysis, i.e., DNA- or isotope analysis [2, 6–8].

Photoluminescence includes fluorescence and phosphorescence. Fluorescence is directly halted when the excitation light ceases, while phosphorescence continues emitting light after the excitation light has ceased. The difference between fluorescence and phosphorescence lies therefore in the decay time, which is in the range of nanoseconds for fluorescence versus microseconds and longer for phosphorescence. This difference is caused by differences in returning to the ground state of the excited electrons. Fluorescence is caused by fluorophores that directly return to the ground state during and after excitation has ceased, for this the spin of the electron in the excited state is opposite to that of the electron in the ground state. Phosphorescence is caused by conversion of the excited electron into the first triplet state, and this electron has the same spin as the ground state, and therefore, the return to ground state is prohibited, resulting in longer lifetimes [9]. In literature, the term “fluorescence” is often used for the photoluminescent effect during excitation with light. However, both fluorescence and phosphorescence occur during excitation with light. Therefore, the term “photoluminescence” should be used if differentiation between the two different emission pathways is not possible [3]. For this study, a distinction was made between long-decay phosphorescence, which is visibly observable, and short-decay phosphorescence, which is visibly not observable.

The bone matrix is heterogeneous and consists out of an organic component, mainly type 1 collagen and adipose, and an in-organic component, mainly hydroxyapatite. Organic components and bioapatites, like hydroxyapatite, are known to both phosphoresce and fluoresce [10–13]. The phosphorescence of calcified tissues, including bone, was measured by Hoerman et al. (1964) [11]. Samples were illuminated using light within the range of 296 to 326 nm. The emitted light with wavelengths longer than 415 nm was measured. Their results showed that human bone phosphoresces with a lifetime of 31 ± 2 s [11]. Bachman (1965) measured the fluorescence of human whole bone in a broad band in the range from 440 to 640 nm after excitation with 365 nm and found that the intensity of fluorescence was highest around 440 nm [12]. However, since bone also phosphoresces, it is uncertain whether the results of Bachman can be solely attributed to fluorescence since phosphorescence might interfere.

Although it is known that inorganic components of the bone matrix contribute to the photoluminescent characteristic, the majority is attributed to organic components [3, 11, 12]. On a microscopic level, the intensity of fluorescence is related to the development of bone matrix and progression of mineralization [13, 14]. Thermally altered human skeletal remains luminesce, when excited with a narrow band light source, and a variety of emission bandwidths can be observed. This characteristic greatly depends ontemperature [2, 3]. At this moment, the interpretation of the photoluminescence of thermally altered human skeletal remains is limited to being present or not and whether orange-red luminescence is observable, which was only observed after exposure to temperatures above 800 °C [1, 3]. Further, it remains to be decided whether phosphorescence contributes to the observed photoluminescence.

Being able to visually differentiate between the two emission pathways, namely, fluorescence with short-decay phosphorescence and long-decay phosphorescence, can be important for the interpretation of the photoluminescent properties of heated bone in case this feature is temperature dependent and might then even be useful for estimating the exposure temperature [3, 6]. Due to the fact that it is currently unknown which emission pathway contributes to the observable heterogenous photoluminescence of thermally altered human bones and whether this is related to the exposure temperature, the long-decay phosphorescence of experimentally heated human bone sections was investigated. The goals of this study are to investigate

- if (thermally altered) human bone phosphoresces visibly to the human eye,
- whether the long-decay phosphorescence depends on temperature, exposure duration, surrounding medium, bone type (cortical or periosteal) and skeletal element, and excitation light,
- whether there are differences between photoluminescence (fluorescence and phosphorescence combined) during excitation and long-decay phosphorescence,
- and if the phosphorescent characteristic can aid in estimating the exposure temperature.

In order to achieve the set goals, heat-induced changes of the observable phosphorescent characteristic of human bone were analysed by means of a scoring index and photographically documented.

## Materials and methodology

For this study, two different sample collections were used. The first collection consisted of human bone sections that were experimentally heated. The second collection consisted of a set of cremated human remains recollected after cremation in a crematorium.

### Sample collections, handling, and heating procedure

Fresh human radii, ulnae, humeri, and femora were extracted from six cadavers that were donated to science. The cadaveric material, consisting of 3 males (aged 59, 66, 79) and 3 females (aged 65, 69, 81), was obtained through the body donation program of the Department of Medical Biology, Amsterdam UMC, location AMC, the Netherlands (see ethical and legal standards section below for further details). Bones were manually defleshed and stored in a refrigerator (range 4 to 7 °C, not longer than 4 days) prior to being sectioned and heated. The long bones were sectioned into 4 mm ± 1 mm transverse diaphyseal section, and 50 mm ± 30 mm diaphyseal thick sections and epiphyseal ends by means of a bone handsaw. The bone material was kept wet during sawing to prevent heating due to friction and unwanted aerial bone dust. For this study, 252 transverse and 52 diaphyseal thick sections and epiphyseal ends were used, resulting in a total of 304 bone samples. The bone sections were divided over temperature–durations categories in the range from room temperature to 1100 °C for 10 to 30 min and heated accordingly in a preheated muffle oven. Samples were either in direct contact with air or covered under a layer of subcutaneous adipose tissue which resulted in complete submersion in adipose at temperatures exceeding the melting point and thereby limiting the oxygen availability, mimicking the presence of soft tissue; see the electronic supplement material (ESM) Section A (Tables s1 and s2) for an overview of the sample sizes per heating subcategory. Samples were then left to cool down to room temperature outside the oven.

In addition, the cremated remains of one cadaver (female, 77 years old) were recollected after cremation at a crematory. The cremation was carried out at 1000 °C for 2.5 h. From the cremated remains, 14 long bone fragments and 10 cranial bone fragments were sorted out. These bones were selected because of their abundant presence and sample quality related to surface intactness.

All samples were handled with tweezers and crucible tongs, and nitrile gloves were worn to prevent contamination during these and subsequent handlings.

### Illumination

Bone samples were placed in a black box, internally covered with non-luminescent black fabric, with an opening on the left side for the alternate light source (ALS, Crime Lite 2, Foster Freeman) and an opening in the front to visually inspect and photograph the samples. Along with the bone section, under investigation, two accompanying bone samples were placed within the black box that served as controls (one bone sample that phosphoresced and one bone sample that did not phosphoresce, and these were selected based on a preliminary investigation of the transverse sections). Bone sections were illuminated in the black box with an ALS emitting either ultraviolet (UV) light in the range from 350 to 380 nm (peak at 365 nm) or blue light ranging from 420 to 470 nm (peak at 445 nm) [3, 15]. The benefit of UV light for this application is that it is not visible to the human eye and, therefore, easier to use since the excitation light does not impede the observer, while the blue light was found to provide the highest intensity of photoluminescence; however, it is less convenient to use due to the intensity of the visible excited light that has to be blocked from the observer to prevent impediment [3]. The cortical surface of the transverse sections and the periosteal surface of the diaphyseal sections and epiphyseal ends were illuminated. Samples were analysed for photoluminescence during illumination and illuminated for approximately 30 s and, directly after turning off the ALS, visually inspected for phosphorescence.

### Phosphorescence index, analysis, and photographic documentation

The intensity of the long-decay phosphorescence of the sample was compared with the positive control bone sample, which exhibited long-decay phosphorescence and were scored by means of the following scoring index, based on previous research: present [strong] (3) if the sample phosphoresced more intensely than the control, present (2) if the sample phosphoresced equally strong as the control, present [weak] (1) if the sample phosphoresced less intensely than the control, and absent (0) in case of no observable phosphorescence [3, 16].

Samples recollected after cremation in a crematorium were analysed while being illuminated (samples illuminated with blue light were studied while wearing a yellow long pass filter goggle, 476nm) and right after illumination on the following features to study differences between photoluminescence and long-decay phosphorescence, derived from previous studies: presence of orange-red luminescence [1, 3], presence of a dark blue or purple luminescent layer [3, 17], and presence of deviating emission bandwidths being higher in intensity than the surrounding bone [3].

Samples were semi-randomly selected for photographic documentation, solely controlled to represent the complete temperature exposure range, and further randomly chosen within the controlled range amongst temperature–duration categories. Photographs were taken with a Nikon D700, equipped with a 35 mm AF-D F2.8 lens or 60 mm AF-D F2.8 micro. Samples were placed on a visually non-luminescent and strongly visible light-absorbing black fabric and photographed in a darkroom. Photographs were taken during illumination for photoluminescence (samples illuminated with blue light were photographed through a yellow long pass filter, Schott GG496 ± 6nm), which applied to samples recollected after cremation only, and directly after turning off the ALS and with a shutter time of 25 to 30 s, diaphragm set at F5.0 to F5.6, and an ISO value of 6400 to 8000, for phosphorescence.

### Statistics

All experimentally heated sections were scored by two observers, TK and LB, at two different moments and in random order without information on the exposure temperature or duration. Inter- and intra-observer agreements were assessed by means of a Kappa test [18]. Based on the suggested levels from McHugh (2012), there was a strong level of agreement for both the intra- and inter-observer scores, see ESM Section B (Tables s3, s4, and s5) for the results of the Kappa analysis [19]. Subsequent analysis was therefore performed on the mean of the four scores; two scores per observer. The mean scores for the transverse sections (4 mm ± 1 mm thickness) were plotted in line graphs, including 2sd. The obtained scores for the diaphyseal sections (50 mm ± 30 mm thickness) and epiphyseal ends were plotted in a scatterplot due to the lower sample size for this collection.

The 14 long and 10 cranial bone fragments, selected from the cremated cadaver, were analysed and scored by TK in the same way as the experimentally heated samples. Of the cranial bones, both the tabula interna and externa were scored, resulting in three groups. The inter-group difference was analysed with a Kruskal-Wallis H test, with significance accepted below 0.05, to test whether different skeletal elements exhibited different intensities of phosphorescence after exposure to heat under similar circumstances.

## Results

### Phosphorescence of thermally altered thin transverse cross sections (cortical bone) heated in air and adipose

**Fig. 1:**
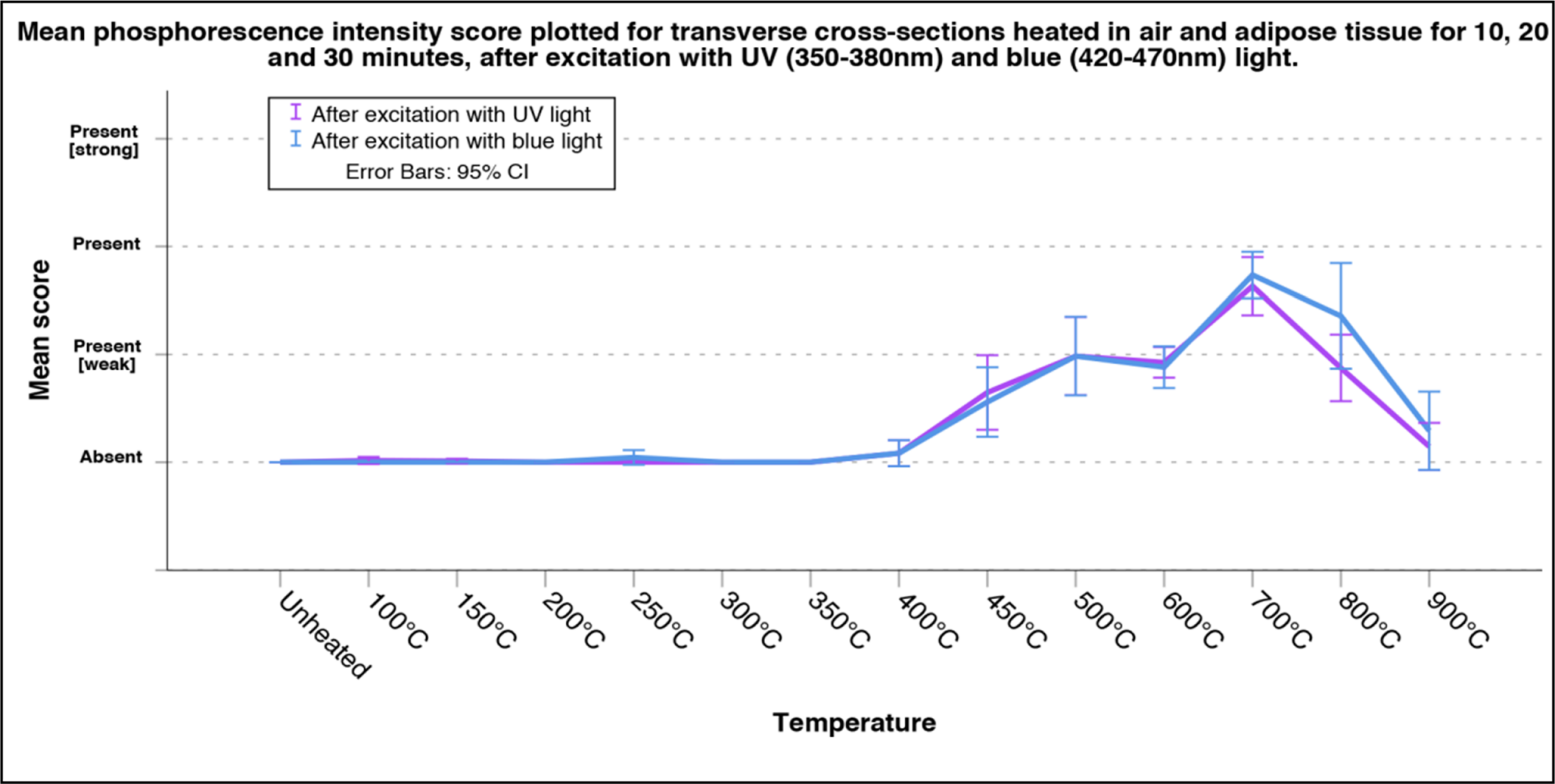
Graph of the obtained overall mean score of the observed phosphorescence after excitation with UV and blue light for the increasing temperature categories which includes exposure duration for 10, 20, and 30 min, based on transverse cross sections of approximately 4 mm heated in both air (up to 900 °C) and adipose (up to 450 °C), total *N* = 252. For individual plots of exposure duration and surrounding medium see ESM Section C, Figs. s1–s4

**Fig. 2:**
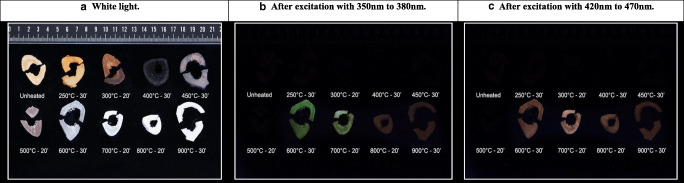
Ten thin transverse sections, heated under controlled circumstances in air within the range of 250 to 900 °C for 30 min including an unheated sample, photographically documented under **a** white light, **b** after excitation with UV light, and **c** after excitation with blue light

The unheated transverse cross sections did not exhibit any phosphorescence after excitation with either UV or blue light. Phosphorescence was scored as absent for the majority of the samples heated to a temperature of up to 400 °C. In the range from unheated to 300 °C, an occasional sample that was heated in medium air exhibited a very faint amount of phosphorescence; however, the decay time was shorter than samples heated to higher temperatures. Samples heated to 400 °C showed small phosphorescent patches that were slightly lighter in colour, more greyish, than the surrounding bone, which was black due to carbonization. With increasing temperature, an increase in phosphorescence was observed. Samples heated to a temperature of 700 °C exhibited the strongest phosphorescence. Thereafter, the phosphorescence intensity decreased with increasing exposure temperatures of up to 900 °C, and some samples heated to 900 °C did not emit light after excitation. The difference in phosphorescence intensity between the durations of exposure of 10, 20, and 30 min at a specific temperature was low; therefore, data was compiled. The same applies to the difference in intensity of the phosphorescence between samples heated in the different surrounding media. For the individual plots based on both the duration of exposure and the surrounding medium for UV light and blue light, see ESM section C (Figs. s1–s4). Except for the samples heated to 800 °C, there was no observable difference between the intensities of phosphorescence after excitation with UV and blue light; see Fig. 1. However, a difference in colour of the emitted light was observed, changing from greenish to pale-reddish accompanied by a decrease in emission intensity, with increasing temperature from 700 to 800 °C after excitation with UV light. This difference in emission colour was not observed after excitation with blue light; see Fig. 2.

### Phosphorescence of thermally altered diaphyseal thick sections and epiphyses (periosteal bone) heated in air

**Fig. 3:**
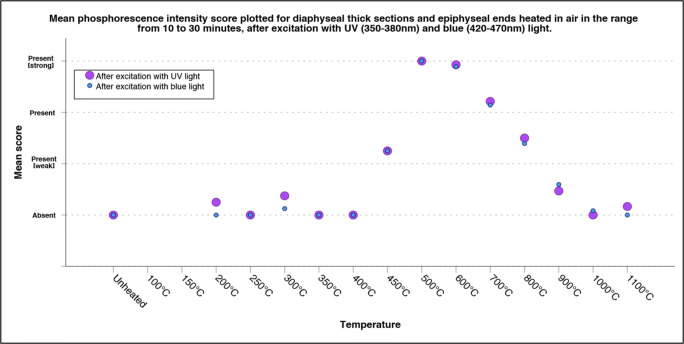
Scatter plot of the mean score of the observed phosphorescence after excitation with UV and blue light for the increasing temperature categories which includes exposure duration in the range of 10 to 30 min, based on diaphyseal sections of approximately 40 mm and epiphyseal ends heated in air (up to 1100 °C), total *N* = 52. See ESM Section C, Figs. s5 and s6 for the individual scatter plots of the data divided per exposure temperature obtained for both UV and blue light

**Fig. 4:**
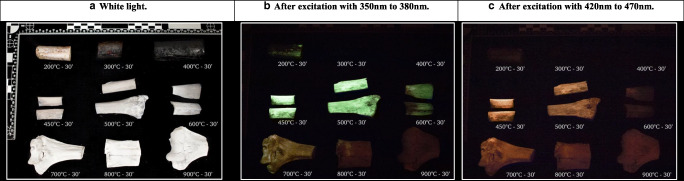
Nine diaphyseal thick sections, heated under controlled circumstances within the range of 200 to 900 °C for 30 min, photographically documented under **a** white light, **b** after excitation with UV light, and **c** after excitation with blue light

Similar to the thin transverse cross sections, the diaphyseal thick sections and epiphyses did not exhibit phosphorescence after exposure to temperatures of up to 400 °C (Fig. 3). A faint phosphorescence with a shorter decay time, similar to the results from the thin transverse sections, was occasionally observed in the range from unheated to 300 °C, and this was also photographically documented (see Fig. 4b for an example). The strongest phosphorescence was observed in the range from 450 to 800 °C, with a maximum obtained for samples heated to 500 °C and 600 °C; see Fig. 3. Again,duration did not have a notable influence on the observed phosphorescence intensity; therefore, data was compiled, and for the individual plots on the exposure duration for both UV light and blue light, see ESM section C (Figs. s5 and s6). A change in colour and intensity of the emission light was observed, resembling the change observed in the thin transverse sections group, after excitation with UV light, with increasing temperature from 600 to 800 °C; see Fig. 4.

### Phosphorescence of the remains collected after cremation and differences between photoluminescence and long-decay phosphorescence

**Fig. 5:**
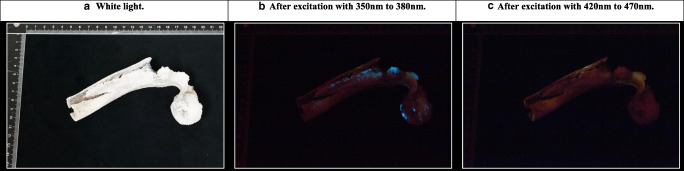
Proximal 1/3th femoral section, collected after cremation by a crematory, photographically documented under **a** white light, **b** after excitation with UV light, and **c** after excitation with blue light

**Fig. 6:**
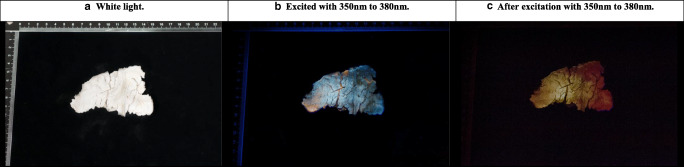
Endocranial (internal table) surface of the occipital bone, collected after cremation by a crematory, photographically documented under **a** white light, **b** under UV light, and **c** after excitation with UV light

The human remains collected after cremation exhibited heterogeneous phosphorescence. Excitation with UV light resulted in blue emission light higher in intensity than the surrounding bone, which appears to originate from a superficial and removable layer; see Fig. 5b. This specific emission light was not observed when the bone was excited with blue light as can be seen in Fig. 5c. The cranial bones exhibited a different emission bandwidth under illumination compared with after illumination; see Fig. 6. The orange-red luminescence, observed inside the heat-induced cracks while being illuminated, was not observed after the light source was turned off, and these area exhibited no to weak intensity of phosphorescence. Further, the blue photoluminescence observable with the light source on was not observed after the light source was turned off; see Fig. 6b and c. A blue or purple luminescent layer was not observed for these samples during or after illumination.

A mean score of 1.4 ± 0.76 was obtained for the long bones, meaning that the intensity ranged from absent to present. The mean score of the cranial bones was 1.6 ± 0.52 for the internal table and 1.5 ± 5.3 for the external table, meaning that the intensity ranged from present weak to present. There was no significant difference between the three groups based on the Kruskal-Wallis test (chi-squared 0.225, df 2, asymp. Sig. 0.894).

## Discussion

Within the investigated sample collection, none of the unheated samples showed long-decay phosphorescence, and this differs from the results of Hoerman et al. who did measure phosphorescence of unheated human bone [11]. The discrepancy in results might be explained by differences in starting material and methodology. Hoerman et al. did not specify the status of the material and did not provide details regarding pretreatment to prevent denaturation besides grinding it to powder and processing it to potassium bromide discs, while in this study fresh human bone was used without any pretreatment. Possibly, the abundant presence of adipose on and in bones inhibits phosphorescence due to reflectance and absorbance of both the excitation and emission light. This is substantiated by the observation that occasionally patches of weak phosphorescence were observed within the range of 100 to 300 °C for 10 to 30 min. Due to heat, in combination with gravity, adipose seeps out of the bone matrix, and at the same time, the bone tissue becomes dehydrated. However, the exact role of adipose and water on the intensity of the emitted light is unknown. Further, the method used by Hoerman et al. is most likely more sensitive than visual inspection as in the current study [11]. The measurements by Hoerman et al. were done at a temperature of − 180 °C in contrast to the current study which was carried out at room temperature (between 18 and 20 °C). The phosphorescence intensity is dependent on the temperature at which the sample was inspected. A lower temperature extends the decay time which will improve the observed and measured intensity of phosphorescence [20].

The sensitivity of the method used in this study was limited, and this hampers statistical analysis on the effect of the variable duration of exposure. Also, the phosphorescence duration was not investigated due to limitations of the chosen method. However, it is clear from the results that the temperature has a bigger influence on the phosphorescence intensity than the duration of exposure to heat. This result corresponds to the results of the previous studies by Krap et al. on changes in photoluminescence and colour [3, 6]. The effect of the surrounding medium, which served to limit oxygen availability to prevent combustion and enable pyrolysis, was negligible due to the fact that the majority of the samples (up to an exposure temperature of 450 °C) did not phosphoresce intense enough to be scored; however, small differences within the samples were observed between the two media. This deviates from the results on photoluminescence from our previous study, in which the effect of adipose tissue as surrounding medium was noteworthy. The temperature range that adipose, as surrounding medium, applies to, both in the experimental setup and in practice, is limited to 450 °C, since adipose auto-ignites at temperatures of around 450 °C and higher, which leads to an instant and incontrollable increase in temperature. Again, it is possible that adipose inhibits phosphorescence, since none of the samples heated in adipose exhibited any phosphorescence up to a temperature of 300 °C, while some that were heated in air did.

Although the transverse sections of 4 mm ± 1 mm, cortical bone, and the diaphyseal sections of 50 mm ± 30 mm and epiphyses, of which the periosteal surface was studied, showed great similarities in results (see Figs. 1 and 3), a notable difference was observed at a temperature of 500 to 600 °C. While the transverse sections in general exhibited weak phosphorescence, the larger sections exhibited strong phosphorescence. This might be explained by differences in microstructure, bone crystal orientation, and degree of mineralization [21]. Prentice has shown that the intensity of photoluminescence is higher where more mineralization has occurred. Since bone remodelling reduces the degree of mineralization, by introducing fresh bone crystals in to the matrix, it has a negative effect on the intensity of photoluminescence [13, 14]. Further, the degree of mineralization, and thus the photoluminescent property, is dependent on the age of the individual and can be influenced by pathological conditions [13, 22]. The sample population of this study consisted of old adults, based on the age categories of Ubelaker et al. (1994), and it is known that subadults have less mineralized bone [23]. It can be expected that burned remains originating from lower age categories exhibit a lower intensity of phosphorescence. However, the same temperature-dependent changes in both intensity and emission bandwidth, as were observed in the old adult category in this study, are expected for the younger age categories, albeit the dynamic range for the changes in intensity can be smaller. This is an important topic for future study.

The results of the current study show that heated human bone fragments visibly phosphoresce. Based on current literature, it is expected that the majority of the organic structures, like the collagen network, have been mostly burned out of the bone matrix at temperatures exceeding 600 °C [24–27]. Therefore, the observed phosphorescence appears to be also, or perhaps even exclusively, caused by the inorganic component of bone since samples heated to temperatures exceeding 600 °C still exhibited phosphorescence. Since this deviates from the current thought that the organic components of the bone matrix are mainly responsible for photoluminescence [10–12], this finding sheds new light on the matter. The photoluminescent properties of the inorganic components and possible interactions with remnants from organic components should be further investigated. For this study, the terminology short- and long-decay phosphorescence was introduced. The observed orange-red luminescence at temperatures exceeding 800 °C (see Fig. 6b) appears not to be caused by long-decay phosphorescence since it was only observed during illumination; see Fig. 6c [3]. This leaves short-decay phosphorescence and fluorescence as possible causes, a finding that can be useful for identifying the underlying cause of the orange-red luminescence.

As previously mentioned, the sensitivity of this study is limited; however, the results are comparable with the practical situation of visually investigating thermally altered human skeletal remains and therefore applicable. The used excitation wavelengths were limited to the range 350 to 470 nm, and this was based on the results of the previous study on photoluminescence [3]. Considering the results of the present study, this bandwidth should be extended since it is unknown what the effect is of illuminating bone beyond this range. Further, besides the limited sensitivity, the dynamic range of the visual scoring index is limited as well. Lastly, the spectral data obtained from the ALSs showed that the output of the ALSs exceeded the ranges as provided by the manufacturer (see ESM Section B, Fig. 1a and c, of Krap et al. 2017) [3]. This results in a decrease in the accuracy of the provided excitation ranges in the present study. The effects are most likely negligible for practice; nonetheless, these findings should be further investigated by means of spectroscopic analysis. However, the observed characteristics were not visible under white light, substantiating the usage of alternate light sources during the laboratory analysis of thermally altered human skeletal remains.

The results of this study show that the phosphorescence emission in the range of 700 to 800 °C changes, which is accompanied by a decrease in intensity, and this is only visible after illumination with UV light. This heat-induced change is related to changes in the bone matrix. At this moment, it is unclear which component(s) contribute to the observed phosphorescence and how these changes relate to the observed heat-induced change. The temperature range in which this heat-induced change occurs is associated with decomposition, calcination, and recrystallisation [7, 28–30]. Since the “greenish” colour of phosphorescence diminishes with increasing temperature, it is more likely an organic molecular component that is thermally broken down than that it is an effect of increasing recrystallisation that occurs. Although organic components like collagen have been shown to endure high temperatures, it is expected that these are not abundantly present and not in their original state and certainly not at temperatures exceeding 600 °C. Another organic molecular component that is broken down at temperatures exceeding 700 °C, and is therefore a possible contributor, is calcium carbonate [24, 31]. The superficial and removable layer that exhibited blue phosphorescence after illumination with UV light might be a surface contaminant; however, it can also be caused by end or byproducts of thermolysis that have separated from the crystalline structure of the hydroxyapatite. The blue phosphorescent layer was only observed within the cremation sample set and not within the experimentally heated set, and differences lie in exposure duration, amount of soft tissue present, and the heating process including handling and transport. Based on the differences, the blue phosphorescence is more likely caused by a surface contaminant; however, more research is needed to substantiate this hypothesis.

Despite the knowledge gap on the origin of the long-decay phosphorescence and subsequent heat-induced changes, the observable changes should be useable to obtain more information on the temperature that the bone has reached, especially considering the strong inter- and intra-observer agreements. However, these results should be validated for different heating contexts and contact surfaces and for the effect of remnants of clothing prior to applying these findings in practice. Furthermore, it is important to compare unknown samples with samples from a proper reference set when analysing these characteristics.

## Conclusion

Thermally altered human skeletal remains have phosphorescent properties. The visibly observable long-decay phosphorescence intensity and emission bandwidth mostly depend on the exposure temperature and excitation bandwidth and less on the exposure duration and surrounding medium during heat exposure. Regarding the temperature that bone tissue can attain during heating, it should be possible to differentiate between temperatures above and below 800 °C by means of assessing the emission bandwidth. Bone exposed to temperatures exceeding 900 °C did not exhibit phosphorescence. The observed phosphorescence coincides with fluorescence and short-decay phosphorescence and thus interferes. The orange-red luminescence that occurs after exposure to temperatures exceeding 800 °C was only observed while being illuminated, leading to the conclusion that this is not caused by long-decay phosphorescence. There were no significant differences in the observed intensity of phosphorescence between cranial (tabula interna and externa) and long bones. Heat-induced changes of phosphorescence intensity of the periosteal surface precede changes in cortical bone, and this can be attributed to differences in structure, density, and possibly presence of molecular remnants or contaminants. UV light provides more information on heat-induced changes of phosphorescence than blue light. Regarding the temperature that bone tissue can attain during heating, it should be possible to differentiate between temperatures above and below 800 °C by means of assessing the emission bandwidth. Spectroscopic analysis is necessary to further investigate the heat-induced changes of the fluorescent and phosphorescent characteristics and to study the relation between the temperature-dependent excitation and emission bandwidths for both pathways.

## Supplementary information

ESM 1 (DOCX 1222 kb).
