# Supplementary material for: Phosphorescence of thermally altered human bone
Source: Int J Legal Med. 2020 Nov 19;135(3):1025–34. doi: 10.1007/s00414-020-02455-1 (PMC8036218; doi:10.1007/s00414-020-02455-1)
Supplement: Supplementary file 1 — (DOCX 1222 kb). [file 414_2020_2455_MOESM1_ESM.docx]

**Electronic Supplement Material**

**Phosphorescence of thermally altered human remains**

*International Journal of Legal Medicine*

Tristan Krap ^1,2,3,4^, Loes Busscher ^4,5^, Roelof-Jan Oostra ^2^, Maurice C.G. Aalders ^5,6^, Wilma Duijst ^1,3^

1. Maastricht University, Maastricht, The Netherlands
2. Amsterdam UMC, Location AMC, department of Medical Biology, section Anatomy, Amsterdam, The Netherlands
3. Ars Cogniscendi Foundation for Legal and Forensic Medicine, Wezep, The Netherlands
4. Department of Life Sciences and Technology–Biotechnology–Forensic Science, Van Hall Larenstein, University of Applied Sciences, Leeuwarden, The Netherlands
5. Amsterdam UMC, Location AMC, department of Biomedical Engineering and Physics, Amsterdam, The Netherlands
6. Co van Ledden Hulsebosch Center, Amsterdam, The Netherlands

Corresponding author:

Name: Tristan Krap

Email: [T.Krap@amc.nl](mailto:T.Krap@amc.nl)

**A: Details concerning sample size**

Table s1 shows the heating scheme and corresponding sample sizes for the transverse cross sections, and table s2 shows the heating scheme and corresponding sample sizes for the thick diaphyseal sections and epiphyseal ends.

Table s1. Heating scheme for the transverse cross-sections, including the surrounding media (N=168 for air, N=84 for adipose tissue).

| **Temperature:** | **Surrounding medium:** | **Duration:** | **N (sample):** |
| --- | --- | --- | --- |
| Unheated | - | - | 8 |
| 100°C | Air  Adipose tissue | 10/20/30 minutes  10/20/30 minutes | 2/2/4  2/2/4 |
| 150°C | Air  Adipose tissue | 10/20/30 minutes  10/20/30 minutes | 4/4/6  2/2/6 |
| 200°C | Air  Adipose tissue | 10/20/30 minutes  10/20/30 minutes | 4/4/10  6/6/10 |
| 250°C | Air  Adipose tissue | 10/20/30 minutes  10/20/30 minutes | 2/2/4  2/2/5 |
| 300°C | Air  Adipose tissue | 10/20/30 minutes  10/20/30 minutes | 2/2/4  2/2/4 |
| 350°C | Air  Adipose tissue | 10/20/30 minutes  10/20/30 minutes | 2/2/4  2/2/4 |
| 400°C | Air  Adipose tissue | 10/20/30 minutes  10/20/30 minutes | 2/4/4  3/4/4 |
| 450°C | Air  Adipose tissue | 10/20/30 minutes 10/20/30 minutes | 2/2/5  2/2/4 |
| 500°C | Air | 10/20/30 minutes | 5/5/7 |
| 600°C | Air | 10/20/30 minutes | 5/5/7 |
| 700°C | Air | 10/20/30 minutes | 5/5/7 |
| 800°C | Air | 10/20/30 minutes | 5/5/4 |
| 900°C | Air | 10/20/30 minutes | 6/2/4 |
| Total: | | | 252 |

Table s2. Heating scheme for the diaphyseal thick sections and epiphyseal ends in medium air (N=52).

| **Temperature:** | **Surrounding medium:** | **Duration:** | **N (sample):** |
| --- | --- | --- | --- |
| Unheated | Air | - | 1 (diaphysis) |
| 200°C | Air | 30 minutes | 2 (1 diaphysis / 1 epiphysis) |
| 250°C | Air | 30 minutes | 1 (diaphysis) |
| 300°C | Air | 30 minutes | 2 (diaphysis) |
| 350°C | Air | 10/20/30 minutes | 2/1/1 (2 diaphysis / 2 epiphysis) |
| 400°C | Air | 10/20/30 minutes | 2/2/2 (4 diaphysis / 2 epiphysis) |
| 450°C | Air | 30 minutes | 2 (diaphysis) |
| 500°C | Air | 30 minutes | 2 (diaphysis) |
| 600°C | Air | 10/20/30 minutes | 2/2/3 (5 diaphysis / 2 epiphysis) |
| 700°C | Air | 10/20/30 minutes | 2/2/3 (5 diaphysis / 2 epiphysis) |
| 800°C | Air | 10/20/30 minutes | 2/2/2 (5 diaphysis / 1 epiphysis) |
| 900°C | Air | 10/20/30 minutes | 3/2/2 (3 diaphysis / 4 epiphysis) |
| 1000°C | Air | 30 minutes | 2 (diaphysis) |
| 1100°C | Air | 10/30 minutes | 1/2 (2 diaphysis / 1 epiphysis) |
| Total: | | | 52 |

**B: Intra- and inter-observer error.**

This section provides details concerning the obtained Kappa tests for the scores of the two observers (TK and LB) based on all samples. Table 3 shows the intra-observer error for the two observers and table 4 shows the inter-observer error between both observers for both scores.

Table s3. Intra-observer error, Kappa agreement value based on the first versus the second score of the observers.

|  | | Symmetric Measures: First versus second score TK – UV light | | | | Symmetric Measures: First versus second score LB – UV light | | | |
| --- | --- | --- | --- | --- | --- | --- | --- | --- | --- |
|  |  |  |  |  |  |  |  |  |  |
|  |  | Value | Asymp. Std. Error^a^ | Approx. T^b^ | Approx. Sig. | Value | Asymp. Std. Error^a^ | Approx. T^b^ | Approx. Sig. |
| Measure of Agreement | Kappa | 0.846 | 0.030 | 21.275 | 0.000 | 0.946 | 0.019 | 22.717 | 0.000 |
| N of Valid Cases | | 304 |  |  |  | 304 |  |  |  |
|  | | Symmetric Measures: First versus second score TK – blue light | | | | Symmetric Measures: First versus second score LB – blue light | | | |
|  | | Value | Asymp. Std. Error^a^ | Approx. T^b^ | Approx. Sig. | Value | Asymp. Std. Error^a^ | Approx. T^b^ | Approx. Sig. |
| Measurment of Kappa Agreement N of Valid Cases | | 0.881  304 | 0.027 | 22.316 | 0.000 | 0.934  304 | 0.020 | 23.057 | 0.000 |
| a. Not assuming the null hypothesis. | | | | | | | | | |
| b. Using the asymptotic standard error assuming the null hypothesis. | | | | | | | | | |

Table s4. Inter-observer error, Kappa agreement value based on the scores between observers after excitation with UV light.

|  | | Symmetric Measures: First score TK versus first score LB | | | | | Symmetric Measures: First score TK versus second score LB | | | |
| --- | --- | --- | --- | --- | --- | --- | --- | --- | --- | --- |
|  |  |  |  |  |  |  |  |  |  |  |
|  |  | Value | Asymp. Std. Error^a^ | | Approx. T^b^ | Approx. Sig. | Value | Asymp. Std. Error^a^ | Approx. T^b^ | Approx. Sig. |
| Measure of Agreement | Kappa | 0.879 | 0.026 | | 21.582 | 0.000 | 0.863 | 0.028 | 21.158 | 0.000 |
| N of Valid Cases | | 304 |  | |  |  | 304 |  |  |  |
|  | | Symmetric Measures: Second score TK versus first score LB | | | | | Symmetric Measures: Second score TK versus second score LB | | | |
|  |  |  |  |  |  |  |  |  |  |  |
|  |  | Value | Asymp. Std. Error^a^ | Approx. T^b^ | | Approx. Sig. | Value | Asymp. Std. Error^a^ | Approx. T^b^ | Approx. Sig. |
| Measure of Agreement | Kappa | 0.809 | 0.032 | 20.027 | | 0.000 | 0.806 | 0.032 | 19.929 | 0.000 |
| N of Valid Cases | | 304 |  |  | |  | 304 |  |  |  |
| a. Not assuming the null hypothesis. | | | | | | | | | | |
| b. Using the asymptotic standard error assuming the null hypothesis. | | | | | | | | | | |

Table s5. Inter-observer error, Kappa agreement value based on the scores between observers after excitation with blue light (420-470nm).

|  | | Symmetric Measures: First score TK versus first score LB | | | | | Symmetric Measures: First score TK versus second score LB | | | |
| --- | --- | --- | --- | --- | --- | --- | --- | --- | --- | --- |
|  |  |  |  |  |  |  |  |  |  |  |
|  |  | Value | Asymp. Std. Error^a^ | | Approx. T^b^ | Approx. Sig. | Value | Asymp. Std. Error^a^ | Approx. T^b^ | Approx. Sig. |
| Measure of Agreement | Kappa | 0.823 | 0.031 | | 20.715 | 0.000 | 0.795 | 0.033 | 19.944 | 0.000 |
| N of Valid Cases | | 304 |  | |  |  | 304 |  |  |  |
|  | | Symmetric Measures: Second score TK versus first score LB | | | | | Symmetric Measures: Second score TK versus second score LB | | | |
|  |  |  |  |  |  |  |  |  |  |  |
|  |  | Value | Asymp. Std. Error^a^ | Approx. T^b^ | | Approx. Sig. | Value | Asymp. Std. Error^a^ | Approx. T^b^ | Approx. Sig. |
| Measure of Agreement | Kappa | 0.805 | 0.032 | 20.227 | | 0.000 | 0.824 | 0.031 | 20.650 | 0.000 |
| N of Valid Cases | | 304 |  |  | |  | 304 |  |  |  |
| a. Not assuming the null hypothesis. | | | | | | | | | | |
| b. Using the asymptotic standard error assuming the null hypothesis. | | | | | | | | | | |

**C. Individual plots.**

Figures s2 and s3 show that there is similarity in phosphorescence intensity between 10, 20 and 30 minutes based on overlap of the 95% confidence intervals. This data is, therefore, combined in one group and shown in a single line plot accompanied by error bars reflecting 95% confidence interval in the manuscript (fig 1). Figures s4 and s5 show that the data of the samples heated in adipose shows great similarity with the data obtained from samples heated in air (figures s2 and s3), therefore this data was also combined with the data on the samples heated in air and shown in figure 1 of the manuscript.

The data obtained from the diaphyseal thick sections and epiphyseal ends, shown in fig s6 and s7 for both UV and blue light, show a trend that is similar to the data of the transverse cross sections in fig 1 of the manuscript, duration of exposure appears to have a limited effect. Therefore, the scores for 10, 20 and 30 minutes were combined in a single group and shown in a scatter plot in the manuscript (fig. 3).

Figure s1. Graph of the mean score of phosphorescence intensity after excitation with UV-light of transverse cross-sections heated in air and adipose tissue plotted per exposure duration of 10, 20, and 30 minutes.

**
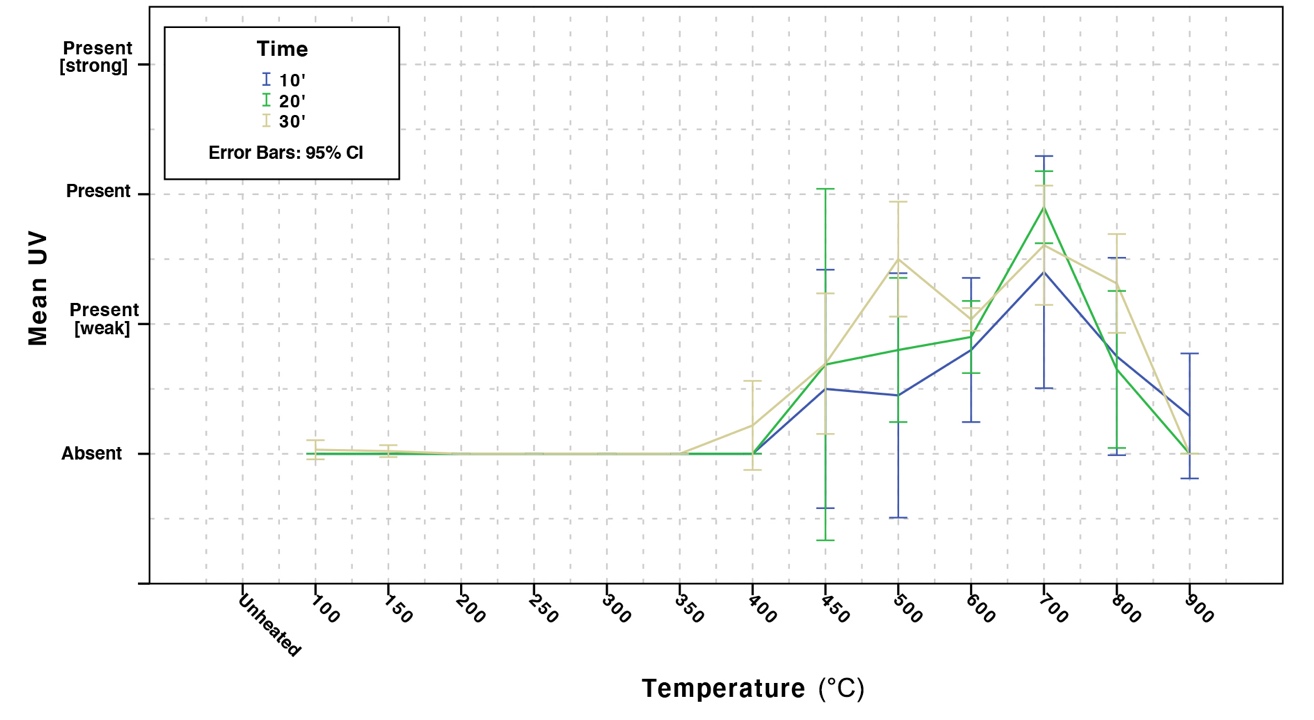
**

**‘ : symbol for minute.**

Figure s2. Graph of the mean score of phosphorescence intensity after excitation with blue light of transverse cross-sections heated in air and adipose tissue plotted per exposure duration of 10, 20, and 30 minutes.

**
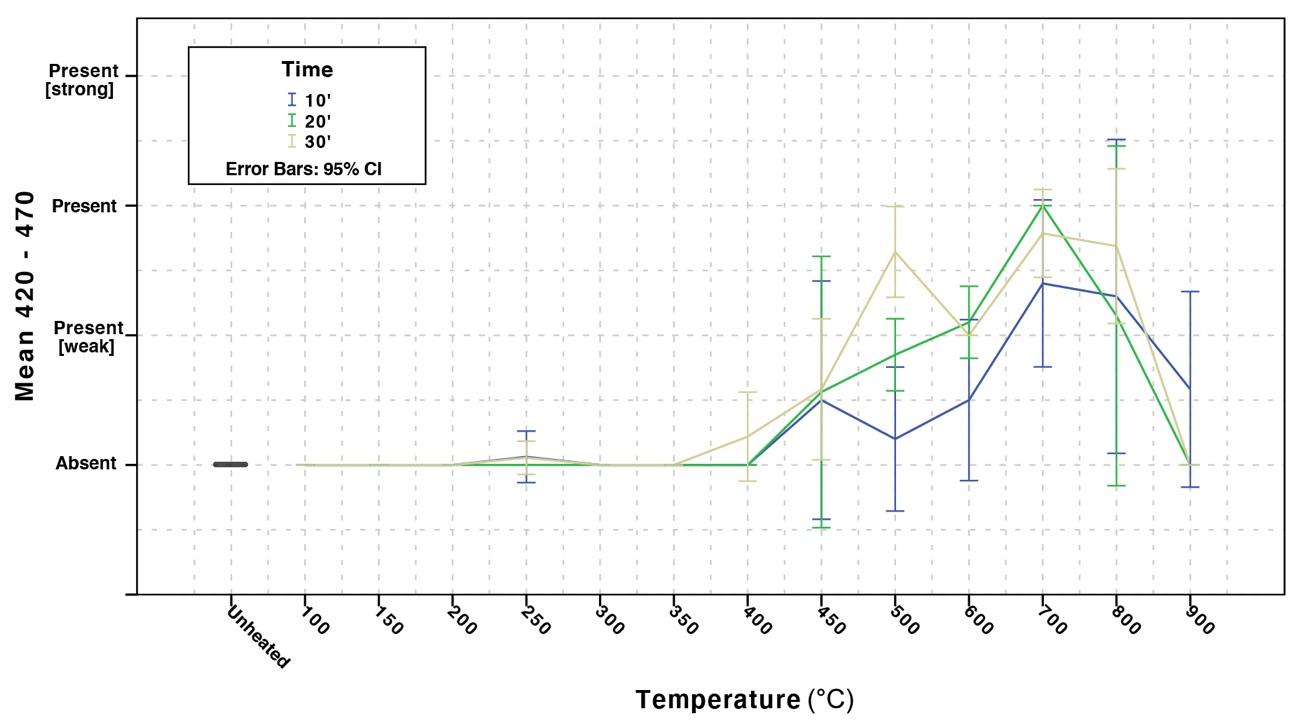
**

**‘ : symbol for minute.**

Figure s3. Graph of the mean score of phosphorescence intensity after excitation with UV-light of transverse cross-sections heated in either air or adipose tissue for 10, 20, and 30 minutes up to 450°C.

**
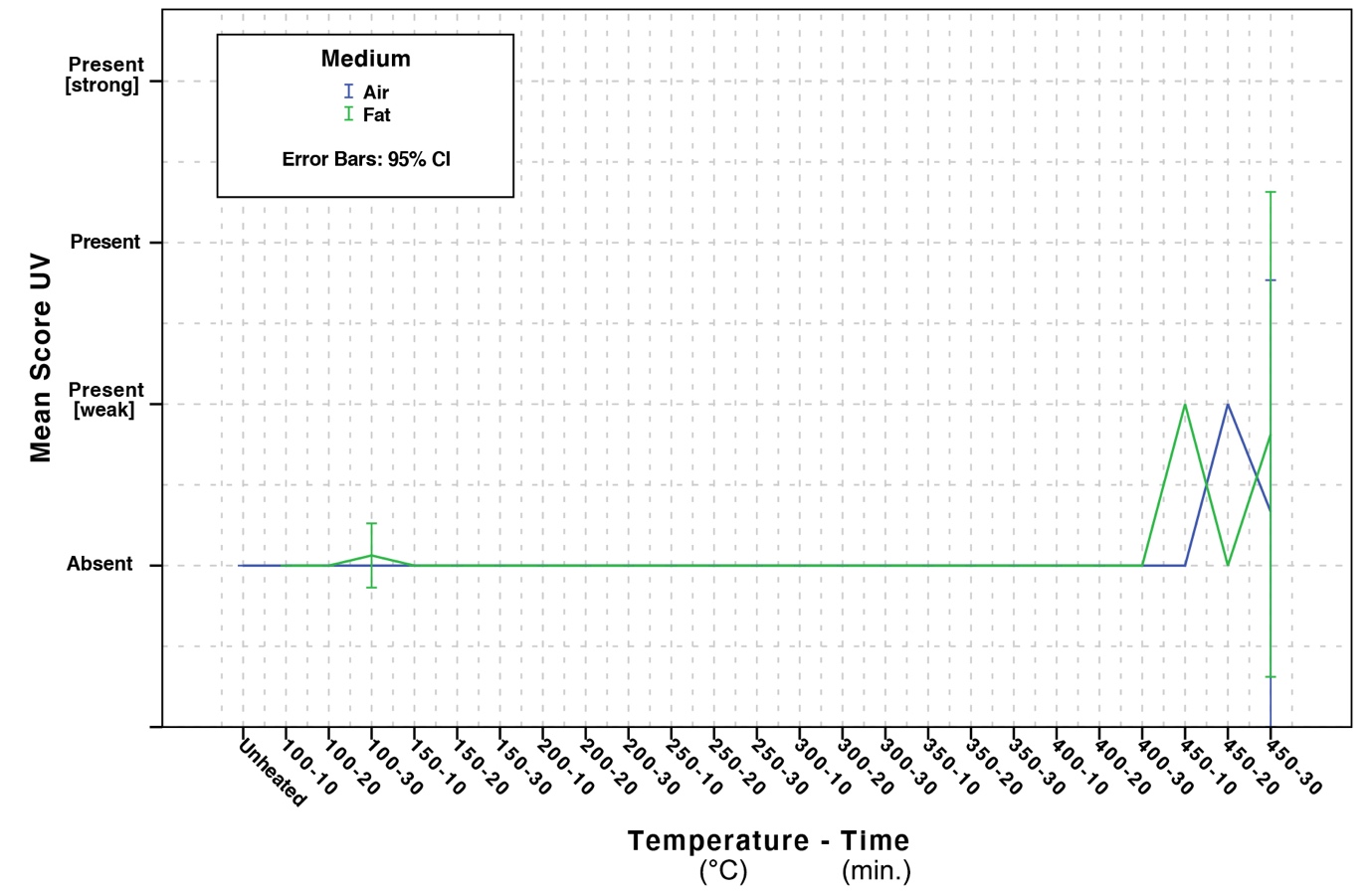
**

Figure s4. Graph of the mean score of phosphorescence intensity after excitation with blue light of transverse cross-sections heated in either air or adipose tissue for 10, 20, and 30 minutes up to 450°C.

**
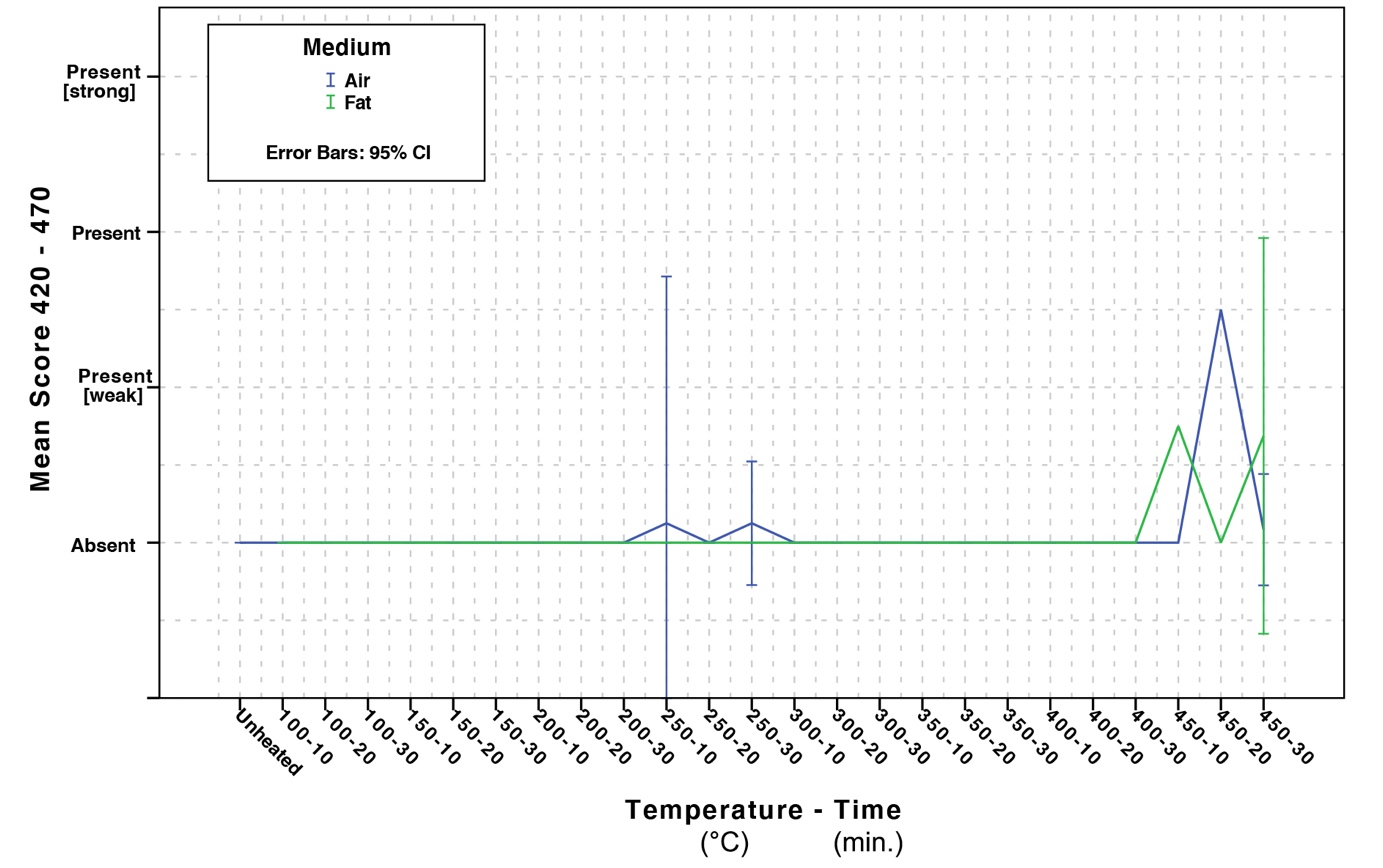
**

Figure s5. Graph of the mean score of phosphorescence intensity after excitation with UV-light of diaphyseal thick sections and epiphyseal ends, heated in air for 10, 20, or 30 minutes.


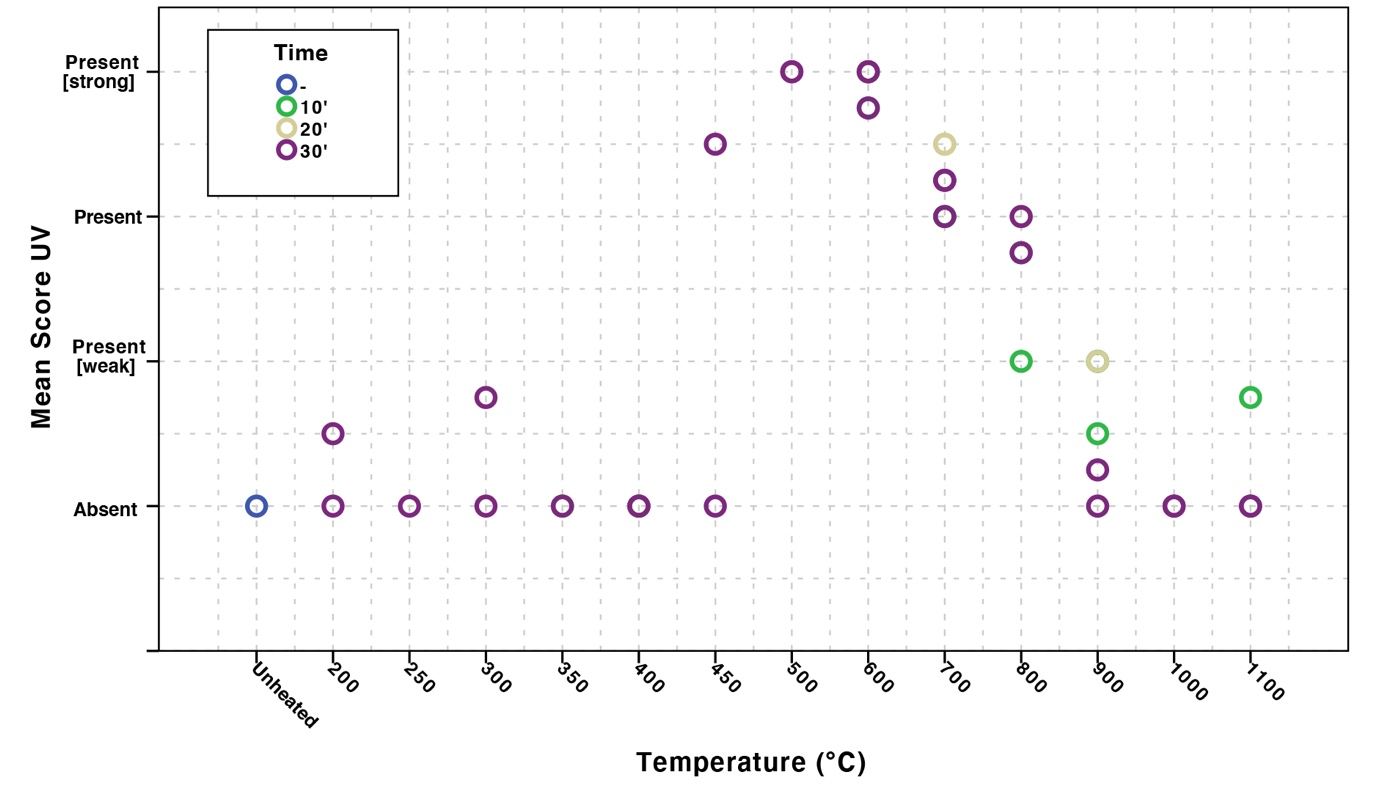


**‘ : symbol for minute.**

Figure s6. Graph of the mean score of phosphorescence intensity after excitation with blue light of diaphyseal thick sections and epiphyseal ends, heated in air for 10, 20, or 30 minutes.


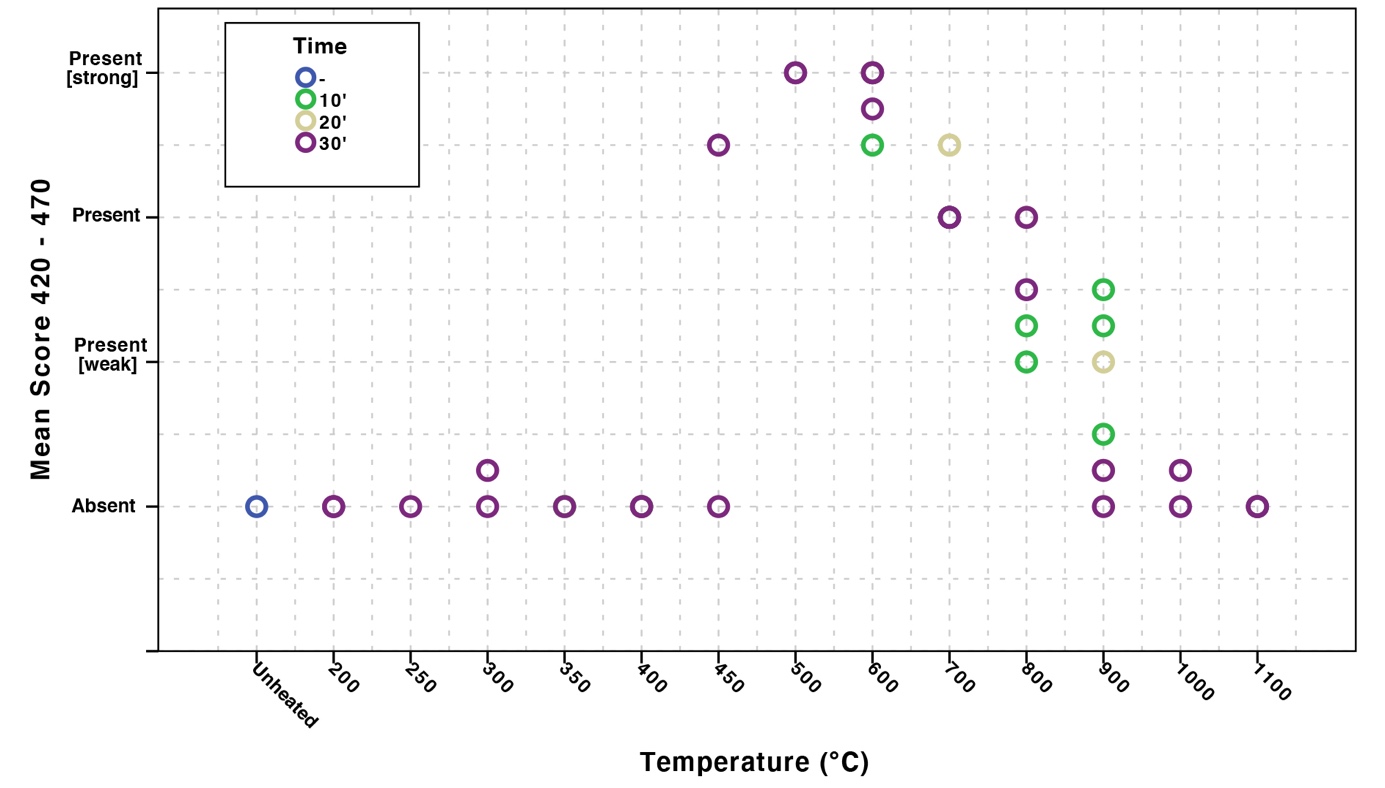


**‘ : symbol for minute.**
